# Neurophysiological Biomarkers in Schizophrenia—P50, Mismatch Negativity, and TMS-EMG and TMS-EEG

**DOI:** 10.3389/fpsyt.2020.00795

**Published:** 2020-08-07

**Authors:** Helena K. Kim, Daniel M. Blumberger, Zafiris J. Daskalakis

**Affiliations:** ^1^Department of Psychiatry, University of Toronto, Toronto, ON, Canada; ^2^Temerty Centre for Therapeutic Brain Intervention, Centre for Addiction and Mental Health, Department of Psychiatry, University of Toronto, Toronto, ON, Canada

**Keywords:** P50, mismatch negativity, schizophrenia, biomarker, early sensory processing

## Abstract

Impaired early auditory processing is a well characterized finding in schizophrenia that is theorized to contribute to clinical symptoms, cognitive impairment, and social dysfunction in patients. Two neurophysiological measures of early auditory processing, P50 gating (“P50”) and mismatch negativity (MMN), which measure sensory gating and detection of change in auditory stimuli, respectively, are consistently shown to be impaired in patients with schizophrenia. Transcranial magnetic stimulation (TMS) may also be a potential method by which sensory processing can be assessed, since TMS paradigms can be used to measure GABA_B_-mediated cortical inhibition that is linked with sensory gating. In this review, we examine the potential of P50, MMN and two TMS paradigms, cortical silent period (CSP) and long-interval intracortical inhibition (LICI), as endophenotypes as well as their ability to be used as predictive markers for interventions targeted at cognitive and psychosocial functioning. Studies consistently support a link between MMN, P50, and cognitive dysfunction, with robust evidence for a link between MMN and psychosocial functioning in schizophrenia as well. Importantly, studies have demonstrated that MMN can be used to predict performance in social and cognitive training tasks. A growing body of studies also supports the potential of MMN to be used as an endophenotype, and future studies are needed to determine if MMN can be used as an endophenotype specifically in schizophrenia. P50, however, has weaker evidence supporting its use as an endophenotype. While CSP and LICI are not as extensively investigated, growing evidence is supporting their potential to be used as an endophenotype in schizophrenia. Future studies that assess the ability of P50, MMN, and TMS neurophysiological measures to predict performance in cognitive and social training programs may identify markers that inform clinical decisions in the treatment of neurocognitive impairments in schizophrenia.

## Introduction

Impaired auditory processing is a consistent finding in schizophrenia and is thought to lead to failures in one’s ability to interact with the environment, contributing to delusional beliefs, hallucinations, social withdrawal, cognitive dysfunction, and decline in functioning (1–10). These deficits have been extensively studied using event-related potentials (ERP) corresponding to early sensory processing events that occur within milliseconds following the reception of an auditory stimulus (7). Mismatch negativity (MMN) and P50 are ERPs that have been extensively studied to examine auditory processing deficits in patients with schizophrenia (11–14). In addition, transcranial magnetic stimulation (TMS), which is an experimental modality with high test-retest reliability, can be used to explore molecular pathways involved in cortical inhibition that are associated with gating of auditory stimuli (15–18). MMN is evoked when there is a rare interruption in a repetitive sequence of stimuli by an “oddball” stimulus that differs from the original sequence by a specific physical quality, such as duration or pitch (19, 20). Deficits in MMN have been associated with decreased ability to orient to critical events occurring in the surrounding environment as well as impaired detection of sensory information that enables identification of social cues (7). Decrease in MMN amplitude has been reported by multiple studies in schizophrenia using a wide range of deviant stimulus characteristics (21–23). P50 is used to assess sensory gating, which represents inhibition of response to a repetitive stimulus (24). Sensory gating is thought to prevent organisms from receiving an overwhelming amount of information from the environment by minimizing response to redundant and irrelevant stimuli (25). Increased ratio of P50 to the redundant auditory stimulus compared to the original stimulus, termed P50 gating ratio, has been shown in patients with schizophrenia (26–39). GABA_B_ receptor-mediated cortical inhibition, which is thought to underlie sensory gating (40), can also be measured using TMS markers, which were shown to be altered in patients with schizophrenia (41), presenting an alternative method to explore neurological processes involved in early sensory processing deficits (15–18). Dysfunction of such neurophysiological filtering may contribute to clinical symptoms and cognitive dysfunction in schizophrenia (42–44).

Biomarkers can aid the identification of molecular pathways, such as genes, underlying a particular disease and/or act as predictive markers to define interventions. While certain biomarkers can serve both functions, some are best suited for one of these purposes. Endophenotypes are conventionally regarded as biological measures that are thought to be closely related to the genetic variation responsible for causing upstream changes, and thus can help understand the molecular mechanisms underlying pathophysiology (45, 46), where recently proposed endophenotypes in schizophrenia have included EEG markers (47, 48). Predictive biomarkers, on the other hand, are those identified using a different set of criteria, as specified by the Measurement and Treatment Research to Improve Cognition in Schizophrenia (MATRICS) initiative, to aid in the development and selection of interventions, with an emphasis on association with a functional outcome and/or pharmacological response (49). Recent studies have explored EEG and neuroimaging markers as predictors of treatment response (50, 51), demonstrating a growing interest in identifying non-invasive endophenotypes and biomarkers in schizophrenia. Characteristics of an endophenotype and a predictive biomarker are summarized in **Table 1**.

**Table 1:** Characteristics of an endophenotype and a predictive biomarker.

Endophenotype (45, 46)	Predictive biomarker (49)
Heritability	Relationship with a functional outcome
Test-retest reliability	Test-retest reliability
Trait stability (i.e. lack of change with pharmacological interventions or disease progression)	Response to pharmacological agents
Diagnostic specificity	Utility as a repeated measure
Greater prevalence in patients compared to the general population	Practicality and tolerability

MMN and P50 are among the neurophysiological markers that are being considered as potential endophenotypes in this disease (52–54). In addition, as MMN and P50 are markers of neurological processes that are thought to be important for cognitive functioning in patients with schizophrenia, they have also garnered interest as predictive biomarkers to see if an intervention will improve cognition (55–60). Tolerability and practicality of ERP measurements are well understood, but their relationship with cognitive and psychosocial outcomes has yet to be fully elucidated (7, 12). TMS paradigms can also be used as an additional modality to explore deficits that may contribute to sensory abnormalities in schizophrenia by altering cortical inhibition. Therefore, in this review, we aimed to provide an updated overview of studies that have examined the potential of P50, MMN, and TMS paradigms to be used as endophenotypes in schizophrenia, as well as their relationship with psychosocial and cognitive functioning in patients to examine their potential to be used as predictive biomarkers for interventions targeted at these domains.

## Mismatch Negativity

MMN is evoked passively when there is a rare oddball stimulus that is presented as an interruption to a sequence of repetitive identical stimuli that occurs within 50ms after the presentation of the oddball and peaks between 100–200 ms (13, 20). MMN is followed by another ERP component, P3a, that peaks between 250–300 ms that is thought to represent the shifting of attention to the oddball stimulus, and another ERP known as RON that peaks between 400–500 ms that is thought to reflect the reorientation of attention to detect further changes in the stimulus (61, 62). While MMN has been most extensively studied within the oddball paradigm and consistently shown to be decreased (i.e., less negative compared to controls) in different stages of schizophrenia (12, 14, 22, 23, 63–82), P3a, and more recently, RON, have also been shown to be impaired in patients with schizophrenia (81, 83–86).

A large body of studies has demonstrated decreased MMN in schizophrenia using different deviant stimuli, with one study reporting an effect size of 0.99 between patients and healthy controls (19, 22, 23, 67, 87, 88). MMN also has good test-retest ability, with studies reporting moderate to high interclass correlation coefficients (ICC) (22, 58, 89). The evidence for trait stability in MMN is mixed. A recent meta-analysis demonstrated that MMN is stable throughout the different stages of schizophrenia (90), while an older meta-analysis showed that MMN to frequency deviant stimuli correlated with illness duration (23). Also, MMN was shown to vary with nicotine, suggesting changes with pharmacological interventions (91). There are also a number of studies demonstrating that MMN varies with the presence and severity of positive and/or negative symptoms measured by scales such as the positive and negative syndrome scale (PANSS) or the psychotic symptom rating scale (PSYRATS) (66, 75, 85, 87, 92–97), while evidence to the contrary exists as well (22, 23, 70, 74, 78, 81, 98, 99). While many studies have consistently reported decreased MMN in schizophrenia, a meta-analysis by Erickson and colleagues have shown that MMN impairment is also present in patients with bipolar disorder (BD) (90). For heritability, Hall and colleagues reported significant heritability for MMN at 63% and 68% for peak amplitude and mean amplitude, respectively (89). An aforementioned meta-analysis showed that there was a trend for decreased MMN amplitude in relatives of patients (90), which was in agreement with another meta-analysis (100). A recently published study also demonstrated an intrinsic effect of 22q11.2 deletion syndrome, a molecular risk factor for schizophrenia, on MMN (54), suggesting a potential genetic link as well. Therefore, while a growing body of studies is supporting MMN as an endophenotype, the findings are mixed. Replication of these findings in future studies will help further demonstrate if MMN can be used as an endophenotype.

MMN has been consistently shown to correlate with various cognitive processes (9, 63, 101–104). Toyomaki and colleagues (105) reported that MMN amplitude was associated with executive functioning as measured by the Wisconsin Card Sorting Test (WCST), Stroop test and trail making test. Supporting this, verbal executive function and verbal IQ were associated with MMN amplitude in patients with schizophrenia (67) and verbal memory was enhanced in patients with prolonged MMN to frequency deviant stimuli (92). On the other hand, Kawakubo and colleagues reported that lower amplitude of MMN is associated with worse verbal memory but not with executive function in patients with schizophrenia (106). This difference in findings between studies may be because MMN amplitude change to different types of deviant stimuli may represent different changes that occur in the brain (92). For example, MMN amplitude change produced by duration and intensity deviance are identifiable in earlier stages of schizophrenia, while amplitude changes to frequency deviance may become more prominent in later stages of the illness (64, 93). The association between poor performance on cognitive tests and MMN amplitude abnormalities in patients with schizophrenia may have implications for daily functioning, as MMN deficits were found to be correlated with greater errors in identification of environmental sounds that are functionally relevant (19). Also, patients with higher MMN amplitude showed greater improvement after auditory perceptual training exercises, suggesting that MMN may be used as a predictive marker in schizophrenia as well (107). Furthermore, when early auditory processing (EAP) was measured by combining MMN, P3a and RON, it was found that EAP was directly associated with a comprehensive assessment of cognitive functioning as measured by the letter number span test, California verbal learning test, Weschler memory scale, and Penn computerized neurocognitive battery (1).

Poor psychosocial functioning as measured by scales such as the global assessment of functioning scale (GAF) and independent living scale (ILS) is considered to be one of the most consistent findings associated with lower MMN amplitude in schizophrenia (9, 22, 63, 64, 99, 106, 108, 109). Supporting this, patients with greater reductions in MMN amplitude were found to be less likely to live in independent settings (22). Also, using the social and occupational functioning assessment scale, lower MMN amplitude was correlated with lower day-to-day functioning (110). Furthermore, when patients with schizophrenia were asked to perform a series of tasks that measured skills necessary for daily functioning, it was found that peak MMN amplitude differences accounted for a significant portion of the variance in performance (9). Wynn and colleagues (108) also reported that higher MMN amplitude was correlated with independent living and higher social perception, possibly contributing to better ability to function in the real world. Supporting this, when patients with schizophrenia received social skills training for 3 months and were assessed on their total social skill scores using a structured role play test, MMN current density values predicted the degree of improvement in patients (63). Interestingly, when MMN, RON, and P3a measurements were used to quantify the EAP, it was found that EAP had a significant association with functional outcome that was mediated by general cognition or negative symptoms (1), suggesting a link between clinical symptoms, cognitive and psychosocial functioning. On the other hand, one study showed that while P3a, MMN and RON amplitudes were associated with psychosocial functioning as assessed with GAF in patients with chronic schizophrenia, this association was not found in patients with a recent onset (81). Also, a recently published study showed that MMN was not correlated with improvement in community functioning or performance on cognitive tasks after cognitive training (60). (1)

## P50

P50 gating (or P50 suppression; “P50”) is a measure of sensory gating, which is thought to be one of the mechanisms underlying positive symptoms of schizophrenia since failure to gate unnecessary sensory input and consequent sensory overload is thought to contribute to hallucinations (26–30). P50 has been used to study auditory processes in schizophrenia for more than 30 years, where subjects are presented with two auditory stimuli that are separated from each other by 500ms, and the amplitude of the evoked potentials at 50ms and 100ms after each stimulus are classified as P50 and N100, respectively (26, 111, 112). The initial stimulus is termed S1, or conditioning stimulus (C), and the second stimulus is termed S2, or test stimulus (T), where the T/C ratio of less than 50% is typically considered to be normal gating (24, 31).

Patients with schizophrenia are consistently reported to have increased T/C ratios, also known as P50 gating deficits, including in meta-analyses (7, 27, 29, 37, 111, 113, 114). N100 gating has also been reported to be deficient in patients with schizophrenia (44, 115). P50 gating has gathered much attention as a potential endophenotype in part because of a genetic association with a polymorphism in the promotor region of the α7-nicotinic cholinergic receptor gene (*CHRNA7*) (116), which was found to be more common in patients with schizophrenia than controls and associated with gating deficiency (117). P50 also has an established biological basis through activation of the GABA_B_ receptors on glutamatergic afferents, resulting in inhibition of pyramidal neurons from firing, which is thought to be impaired in schizophrenia (40, 118). This suggests, however, that P50 may be affected by pharmacological agents that act through the same receptor and therefore less likely to be a trait marker. Indeed, clozapine was shown to improve P50 gating in patients with schizophrenia, likely related to potentiation of the GABA_B_ receptor (119). It has also been shown that while P50 may not be affected by agents that almost exclusively target dopaminergic neurotransmission, it is affected by atypical antipsychotics, such as clozapine and olanzapine (42, 119, 120). Studies have also demonstrated that P50 gating ratios differ with intensity, severity and frequency of hallucinations (26, 121–124), although the evidence for this is weak, as there are studies demonstrating the opposite as well (30, 120, 125–128). The same was found for negative symptoms, where studies have both demonstrated a lack of association with P50 (24, 30, 39, 120, 125, 126, 129–132) as well as a significant relationship (43, 133). Outside of positive and negative symptoms of schizophrenia, impaired P50 gating was found to be associated with anxiety, depression and anergia in patients who were recently diagnosed with schizophrenia (130). Therefore, evidence suggests that P50 is likely not a trait stable marker, although it is uncertain if it is a state marker. Studies also showed that P50 lacks in diagnostic specificity, as P50 suppression was found in patients with BD, relatives of patients with psychotic BD (134), and Alzheimer’s disease (135, 136). For heritability, a meta-analysis by de Wilde and colleagues showed a moderate to large effect size for P50 in relatives of patients with schizophrenia (114). Twin studies have also reported a significantly higher ICC for monozygotic twins compared to dizygotic twins (137, 138), suggesting heritability, with one study reporting heritability for the T/C ratio to be 68% (89). On the other hand, a study examined 183 nuclear families of patients with schizophrenia and found that heritability was not statistically significant (139). Lastly, P50 has been shown to have low test-retest reliability (58), with ICC being shown to be less than 0.5 in one study (33). However, another study reported that P50 gating ratio had a ICC of 0.66 (89), suggesting that the findings are inconsistent.

The relationship between P50 gating deficiencies and cognitive functioning has been extensively studied (8, 11, 24, 140). Patients with schizophrenia who were classified as having a high gating abnormality according to their P50 gating ratios were found to have impaired sustained attention (30). This was in agreement with several studies that also showed an association between impairment in P50 gating and poor attention, working memory and lower processing speed (24, 26, 30, 121, 130, 141, 142). Furthermore, in one study, P50 and N100 amplitudes were able to predict working memory, attention, and long delayed memory, where variance in attention remained significantly associated with P50 even after correcting for general cognitive ability as measured by IQ (121). Interestingly, one study showed that P50 gating deficiencies were associated with worse performance on the color pattern implicit learning task, suggesting that this gating deficiency may extend to other areas of learning as well (143). The same study demonstrated that P50 gating deficit may have different effects on visual and auditory learning, as one study found that P50 gating impairment was correlated with visual but not with verbal implicit memory test performance (143). In contrast, Cullum and colleagues reported that P50 gating ratio is not correlated with processing speed (26). Using the WCST and Repeatable Battery for the Assessment of Neuropsychological Status (RBANS), three studies also found a lack of relationship between P50 ratio and cognitive functioning (30, 59, 126).

While the relationship between P50 gating and psychosocial or global functioning have not been as extensively examined as for MMN, one study reported that patients with impaired P50 gating have worse community outcome (113), where they were found to have worse GAF scores and lower quality of life scores, suggesting that sensory gating impairments may contribute to functional impairment as well (8, 113).

## TMS-EMG and TMS-EEG Paradigms

TMS uses magnetic fields to induce eddy currents in the cortex through electromagnetic induction (144). It is unique in that it can differentially stimulate inhibitory interneurons and pyramidal neurons, allowing for the measurement of GABA receptor-mediated inhibition (17), which was shown to be impaired in schizophrenia (40, 41, 145). Animal models have shown a direct link between GABA_B_ receptor blockade and impaired sensory gating (146, 147). These findings, together with studies demonstrating the involvement of GABA_B_ activation in sensory gating as measured by P50 in humans (40), suggest that TMS paradigms for measuring GABA_B_ receptor-mediated cortical inhibition can be used to examine sensory gating in schizophrenia (17, 18, 144). Two TMS paradigms that can provide a measure of GABA_B_ receptor inhibition are long interval cortical inhibition (LICI) and the cortical silent period (CSP) (15–17). LICI is analogous to the P50 paradigm, where a suprathreshold conditioning stimulus is followed by a suprathreshold test stimulus by 50–200 ms, producing an inhibition of the motor evoked potential (MEP) by 50% (148). LICI can also be examined using TMS-EEG by measuring P30, which is an early TMS-evoked cortical potential (149). CSP uses stimulation to the motor cortex which, at high intensities, causes cessation of all background EMG activity that is normally ongoing, where the duration of the “silent period” is measured to quantify the extent of GABAergic inhibition (150). EEG indices of CSP have also been explored, where power of alpha and delta oscillations were found to be related to CSP duration (151). Importantly, CSP and P50 were found to be significantly correlated (152), providing additional evidence that they work through the same mechanism (17, 153, 154) Furthermore, CSP was found to be impaired in patients with schizophrenia (41, 155), suggesting that CSP may be a good candidate for the study for GABA_B_ receptor inhibition in schizophrenia, which is implicated in sensory gating deficits in this population. (156)

TMS paradigms have more recently emerged as potential endophenotypes of schizophrenia. Several findings support LICI and CSP as indices of GABA_B_ receptor inhibition, including the ability of baclofen, a GABA_B_ agonist, to increase LICI and CSP duration (153, 154), and the fact that slow inhibitory post-synaptic potentials mediated by GABA_B_ receptors peak at 150s, which is the duration of LICI and CSP (17). These studies support a biological basis for these TMS paradigms. It was also demonstrated that TMS measures have a high test-retest reliability with ICC being greater than 0.8 in the motor cortex (18). One study examining prefrontal cortex LICI in patients with schizophrenia, obsessive compulsive disorder, and healthy controls found that LICI deficit was specific to schizophrenia (157), suggesting diagnostic specificity. For heritability, a lack of difference in LICI was found between first degree relatives of patients with schizophrenia and controls (158), while the heritability of CSP has yet to be examined. One study examining CSP in patients with chronic schizophrenia, recent-onset schizophrenia, and healthy controls reported a significant difference between the three groups, which was driven by the shorter CSP in recent-onset patients compared to health controls (159). In addition to baclofen, CSP was reported to increase with clozapine and quetiapine (160–162), while one study reported no difference in CSP between medicated and un-medicated patients (163). On the other hand, LICI was not found to differ with cannabis use in patients with schizophrenia (164), and transcranial direct current stimulation failed to modulate CSP in patients with schizophrenia (165). While these studies show mixed findings for trait stability, they suggest a potential for CSP and LICI to be used as markers of treatment response with certain medications as it shows modulation with pharmacological agents. TMS is also a non-invasive procedure with high tolerability (166, 167), making it ideal for the use of identification of endophenotypes and/or predictive markers.

CSP and LICI have not been extensively examined for their potential to be used as predictive biomarkers in schizophrenia. One study examined the relationship between LICI and social cognition and did not find a significant correlation (156). While more studies are needed to identify if TMS paradigms can be used as predictive biomarkers for cognitive and social functioning, their high-test retest reliability and clear association with a biological pathway suggest that it may be a promising avenue to explore for the identification of endophenotypes and/or predictive biomarkers for pharmacological agents that may impact sensory processing.

## Discussion

**Figure 1:**
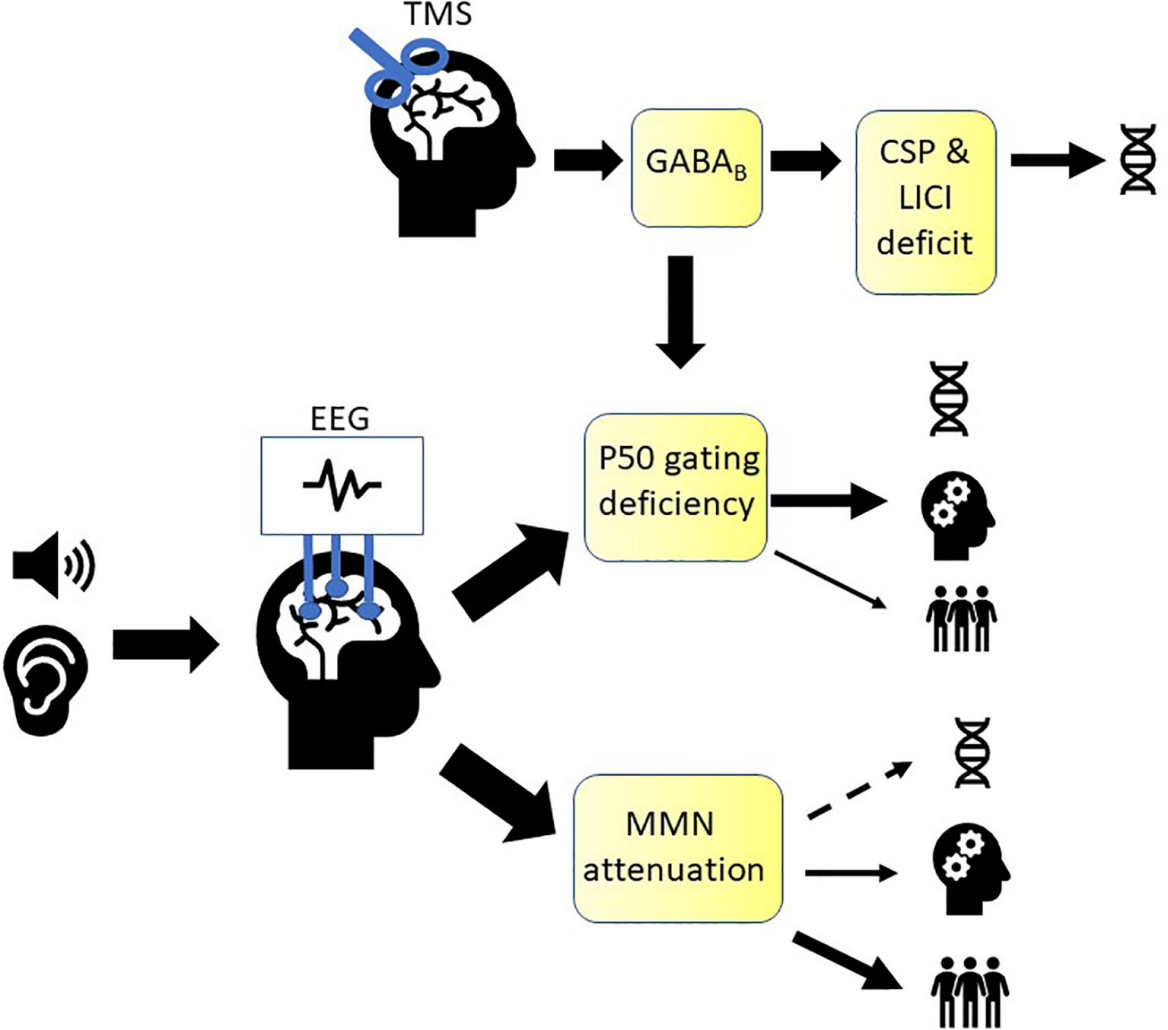
Summary of markers of early auditory sensory processing in schizophrenia. A growing body of evidence is supporting mismatch negativity (MMN) as an endophenotype in schizophrenia (denoted by the DNA icon), while evidence is weaker for P50. Both were shown to be associated with cognitive functioning (denoted by the cogwheel icon) and psychosocial functioning (denoted by the people icon). P50 gating ratio is thought to represent in part GABAB receptor-mediated cortical inhibition, which can also be measured using TMS (transcranial magnetic stimulation) paradigms, cortical silent period (CSP) and long-interval intracortical inhibition (LICI). CSP and LICI are promising candidates for endophenotypes that can be used to study sensory gating in schizophrenia.

Deficits in EAP have been extensively studied in patients with schizophrenia, where they are thought to contribute to failures in detecting important environmental cues and filtering of irrelevant stimuli, possibly leading to social withdrawal, hallucinations, cognitive dysfunction, and decreased level of overall day-to-day functioning (1, 3, 4, 6, 7, 9, 10, 12, 19, 88, 100, 114, 168). Here, we reviewed studies examining if MMN and P50 meet the 5 criteria for a marker to be considered an endophenotype (46), as well as their relationship with cognitive and psychosocial functioning to examine their potential to be used as markers to inform interventions targeted at improving these domains. Furthermore, we examined two TMS paradigms, CSP and LICI, for their potential to be used as markers of sensory gating and as endophenotypes in schizophrenia. A diagram summarizing the findings discussed in this review can be found in **Figure 1**.

Impairment in MMN is consistently demonstrated in patients with schizophrenia (14, 19, 88) with studies showing moderate to high test-retest reliability (58, 89). However, studies examining heritability, trait stability and diagnostic specificity of MMN have reported mixed findings (89, 90, 100) suggesting that while promising, future studies further demonstrating MMN’s suitability as an endophenotype for schizophrenia would be beneficial. A significant correlation between MMN and performance in cognitive tasks is consistently reported in schizophrenia (80). Furthermore, MMN amplitude was able to predict how a patient would perform in auditory perceptual training exercises (107), suggesting its utility as a predictive marker as well. (1, 11). Studies have also consistently shown that lower MMN is correlated with poor psychosocial functioning and ability to live in independent settings (1, 11, 22, 88, 108, 109, 169). Furthermore, MMN was also able to predict performance in social skills training in patients with schizophrenia, again demonstrating its ability to be used as a predictive marker (63), although evidence for the contrary exists as well (60). These studies demonstrate the potential for MMN to be used as a surrogate marker/endpoint and a predictive marker for cognitive and psychosocial dysfunction.

P50 is a marker of sensory gating, which is thought to be an important cognitive process involved in the development of schizophrenia (11, 24, 141). Like MMN, impaired P50 gating is consistently reported in patients with schizophrenia compared to healthy controls (111, 114). While P50 has a strong link to the *CHRNA7* gene and the GABA_B_ receptor (116, 117, 152), the evidence for P50 as an endophenotype of schizophrenia is inconsistent for heritability, test-retest reliability, and heritability (24, 39, 58, 89, 120, 122, 133, 134, 139, 170) suggesting that more studies are required to ascertain if P50 can be used as an endophenotype in schizophrenia. Sensory gating is also thought to play an important role in various cognitive processes (140), and accumulating evidence is supporting an association between P50 gating deficiencies and cognitive deficits (8, 26, 29, 121, 141, 143), although there are studies reporting the contrary (26, 30, 126). Evidence is also accumulating for N100 gating ratio and its potential to act as a marker for cognitive functioning in patients with schizophrenia (44, 58, 112, 121). While the relationship between P50 gating ratio and psychosocial and global functioning has not been extensively examined, (8) one study reported an association between P50 gating ratio deficit and poor community outcome (113). Together, these studies suggest that more studies are needed to elucidate the link between P50 and global functioning, while P50 may hold promise as a marker of cognitive impairment in schizophrenia.

GABA_B_ receptor-mediated cortical inhibition is implicated in sensory gating (147, 171), which can be measured using two TMS paradigms, LICI and CSP (16, 17). Several lines of evidence support LICI and CSP as measures of GABAergic inhibition, including increased duration with a GABA_B_ agonist, baclofen (153, 154). Furthermore, CSP was found to be significantly correlated with P50 (152), suggesting that it may be used as a marker to study GABA_B_ receptor-mediated cortical inhibition in schizophrenia. TMS measures have a high test-retest reliability (18) and accumulating evidence is demonstrating impaired CSP and LICI (41, 155) in patients with schizophrenia, which may be specific to this diagnosis (160). Studies examining trait stability of CSP and LICI report mixed findings (160–165), and more studies are needed to establish heritability of these markers in schizophrenia. Together, these findings suggest that CSP and LICI are promising potential endophenotypes to examine sensory processing deficits in schizophrenia, although more studies are needed (152).

In summary, there is an accumulating body of evidence is supporting a link between P50, MMN and cognitive functioning in schizophrenia. Future studies are needed to assess the ability of P50 and MMN to inform clinical decisions as predictive biomarkers in the treatment of schizophrenia. While MMN is also correlated with global and psychosocial functioning, their association with P50 has not been extensively studied (92, 108, 109). Future studies may benefit from exploring the relationship between P50 and functional outcomes in schizophrenia as well to further understand the contribution of early sensory processing deficits in functional outcomes in this disease. P50 has inconsistent evidence at this time to support its suitability as an endophenotype, suggesting that it may represent more dynamic processes influenced by internal and external changes rather than upstream, genetically linked processes that are stable throughout the course of one’s life. On the other hand, studies have demonstrated potential for MMN to be used as an endophenotype. CSP and LICI may also represent promising biological targets for examining sensory processing deficits in schizophrenia, with accumulating evidence supporting their suitability to be used as endophenotypes as well. Future studies further investigating these markers may yield important findings for the identification of biomarkers in schizophrenia.

## Author Contributions

HK, DB, and ZD took part in the literature review, manuscript writing and revision of the manuscript. All authors contributed to the article and approved the submitted version.

## Conflict of Interest

In the last 5 years, ZD has received research and equipment in-kind support for an investigator-initiated study through Brainsway Inc. and Magventure Inc. His work is supported by the Canadian Institutes of Health Research (CIHR), the National Institutes of Mental Health (NIMH), Brain Canada, and the Temerty Family and Grant Family through the Centre for Addiction and Mental Health (CAMH) Foundation and the Campbell Institute. DB has received research support from CIHR, NIH, Brain Canada, and the Temerty Family through the CAMH Foundation and the Campbell Family Research Institute. He received research support and in-kind equipment support for an investigator-initiated study from Brainsway Ltd. He is the site principal investigator for three sponsor-initiated studies for Brainsway Ltd. He also receives in-kind equipment support from Magventure for two investigator-initiated studies. He received medication supplies for an investigator-initiated trial from Indivior.

The remaining author declares that the research was conducted in the absence of any commercial or financial relationships that could be construed as a potential conflict of interest.
